# On the origin of myeloid-derived suppressor cells

**DOI:** 10.18632/oncotarget.12278

**Published:** 2016-09-27

**Authors:** Camilla Rydberg Millrud, Caroline Bergenfelz, Karin Leandersson

**Affiliations:** ^1^ Department of Translational Medicine, Cancer Immunology, Lund University, Skåne University Hospital, Malmö, Sweden; ^2^ Department of Translational Medicine, Division of Experimental Infection Medicine, Lund University, Malmö, Sweden

**Keywords:** MDSC origin, myelopoiesis, emergency myelopoiesis, reprogramming, extramedullary

## Abstract

Myeloid-derived suppressor cells (MDSCs) have a strong immunosuppressive character that allows them to regulate immune responses and hinder overt inflammatory responses. In cancer, this leads to tumor immune evasion and disease progression. MDSCs come in at least two forms: monocytic (Mo-MDSCs) and granulocytic (G-MDSCs). The classical definition of MDSCs as immature myeloid cells blocked from differentiating has been challenged by recent studies suggesting that Mo-MDSCs and G-MDSCs may represent monocytes and granulocytes that have acquired immunosuppressive properties. The molecular mechanism behind their generation and their true origins are now widely debated. In this review we discuss the different proposed mechanisms of the generation of both types of MDSCs, with a special focus on human MDSCs in cancer.

## INTRODUCTION

The term “myeloid-derived suppressor cells” (MDSCs) was coined in 2007 to describe a non-lymphoid immune suppressor cell population of myeloid origin that was enriched in cancer patients [1]. We now know that MDSCs constitute a population of myeloid cells with heterogeneous morphology, surface phenotype, and function, but with strong immunosuppressive properties in common. These cells are enriched in different pathological conditions including cancer, trauma, and sepsis, with cancer being the predominant condition in which MDSCs have been described [2–5]. Indeed, the elimination of MDSCs dramatically improves immune response in cancer patients and tumor-bearing mice [2, 6, 7].

**Figure 1:**
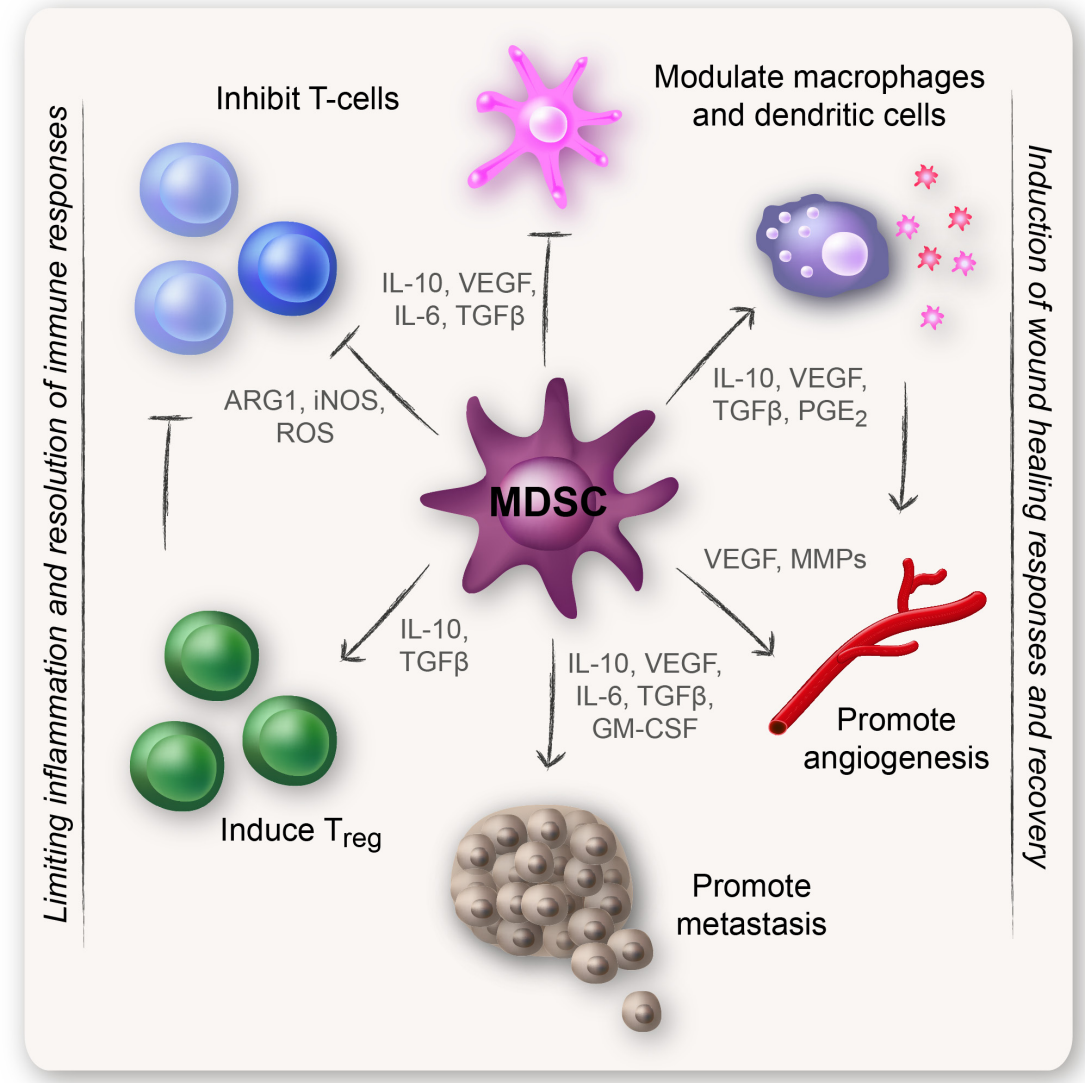
Classical MDSC functions MDSCs have a strong immunosuppressive character that allows them to limit inflammation so that wound healing and recovery can take place. One of the hallmarks of MDSCs is their ability to suppress T-cell responses. MDSCs have also been described to induce regulatory T-cells (Tregs), modulate the cytokine production of macrophages, and promote other non-immune functions such as tumor angiogenesis and eventually metastasis.

MDSCs are an important node in the cellular network that regulates immune responses. One of the hallmarks of MDSCs is their ability to suppress T-cell responses. MDSCs have also been described to regulate innate immune responses by modulating the cytokine production of macrophages [2, 4, 5]. Immunosuppressive myeloid cells have most likely been generated as a normal physiological response to acute and excessive inflammatory conditions during evolution. It is therefore not surprising that MDSCs are present at high numbers in tumors, since tumors show chronic inflammation normally controlled by regulatory immunosuppressive cells. In tumors, MDSCs also promote other non-immune functions such as tumor angiogenesis and eventually metastasis [8–10], perhaps reflecting the natural role of MDSCs during wound healing. Because of their suppression of anti-tumor immune responses, MDSCs are often described as “bad cells.” As such, MDSCs provide a favorable microenvironment in which transformed cells can proliferate, acquire new mutations, expand, and evade host immunosurveillance [2, 4]. Some typical MDSC functions are listed in Figure 1.

The accumulation of MDSCs in cancer patients is a generally accepted phenomenon [11, 12]. Its clinical relevance has also been reported for a substantial number of cancers, in which circulating MDSCs have been correlated with clinical cancer stage and tumor burden in patients with different tumors [13–19]. MDSCs have also been negatively correlated with immune responses to cancer therapy [20, 21]. The regulatory role of MDSCs is nonetheless crucial for limiting inflammation and for resolving immune responses in general, so that wound healing and recovery can take place, thereby restoring homeostasis [22, 23]. MDSCs are also thought to protect the host during severe infections through the regulation of inflammatory responses [24]. Indeed, MDSCs accumulate in acute life-threatening conditions such as sepsis, initially limiting the devastating effect of an excessive inflammatory response, and they even might promote bacterial clearance [25]. However, the high level of MDSCs generated probably also contribute to the potentially fatal immune paralysis observed during the later stages of sepsis [26]. Finally, their ability to suppress T-cells may also serve to prevent the development of autoimmune diseases by dampening inappropriate immune reactions [22, 23]. Although the functional importance of MDSCs as regulatory cells has emerged in recent years, there are still uncertainties about their generation and origins. In this review, we attempt to distinguish the different aspects of and theories on the origin of MDSCs with a focus on cancer.

## MDSC CHARACTERIZATION

In terms of morphology, surface phenotype, and function, MDSCs are not a defined subset of myeloid cells, but rather a heterogeneous population. As such, they express a mixture of surface markers typical for myeloid cells, but lack lineage markers for lymphocytes, natural killer cells, macrophages, and dendritic cells [4, 27–29]. Two major groups of MDSCs have been characterized to date: cells with a morphology and surface phenotype typical for monocytes (Mo-MDSCs) and cells with a surface phenotype typical for granulocytes (G-MDSCs - also called polymorphonuclear [PMN]-MDSCs), but with a heterogeneous morphology including granulocytes, blasts, or cells with ring-shaped nuclei [27–29].

Historically, MDSCs have been regarded as immature cells. The main reasons for this would be their surface phenotype seen using flow-cytometric analysis, their morphology, and their ability to differentiate into macrophages, dendritic cells, or granulocytes [3, 4, 29]. The immature profile could possibly, at least in part, be opened for reevaluation. The surface phenotype does represent immature cells of myeloid origin, since they express myeloid cell lineage markers but lack activation and maturation markers. This view, however, could be criticized because mature cells of the myeloid lineage could also lose activation markers upon repeated toll-like receptor (TLR)-signaling, exposure to certain cytokines [30–34], or in response to hypoxia [35, 36]. Considerable evidence has emerged that Mo-MDSCs and G-MDSCs may even represent monocytes and neutrophils, respectively, that have been reprogrammed or activated into immunosuppressive populations [26, 29].

MDSCs were originally found in mice, and the surface phenotypes differ vastly between mice and humans. In mice, MDSCs are characterized as Gr-1+CD11b+ cells, and further Mo-MDSCs are described as CD11b+Ly6ChighLy6G- cells and G-MDSCs as CD11b+Ly6ClowLy6G+ expressing cells [4, 5, 28]. In humans, the phenotypic characterization of MDSCs has proven difficult. A great number of surface phenotypes have been described, with significant variations between different individuals, indicating that there may be distinct subpopulations of MDSCs besides G-MDSCs and Mo-MDSCs [4, 5, 28]. However, the increasingly accepted - although still debated - definitions of human MDSCs are CD11b+CD14+CD33+HLA-DR-/lowCo-receptor-/low (Mo-MDSCs) and CD11b+CD15+CD33+Lin-HLA-DR-/low (G-MDSCs) expressing cells, present in the mononuclear fraction of density gradients [5, 23, 37].

New potential candidates continue to be found to further characterize the human MDSCs such as CD66b, CD115 (CSF-1R; M-CSF receptor), CD124 (IL-4Rα), CD40, CD80, and S100A9 [4, 5, 23, 37–39]. Although these markers are undoubtedly expressed by MDSCs, they do not define a specific MDSC population with distinct suppressive functions. Given their heterogeneity, the definition and characterization of MDSCs are somewhat controversial and many studies concerning phenotypic characterization of MDSCs in humans have not studied their suppressive function, even though this activity is a mandatory criterion [40–44]. However, in recent years the suppressive function or markers specific for immune suppression (e.g., Arginase-1 [ARG1]) have been added to their phenotype definition [45].

MDSCs can employ a wide range of suppressive mechanisms that often includes more than one single mechanism (Figure 1). In humans, G-MDSCs are mostly known to inhibit T-cells via the production of reactive oxygen species (ROS) [46]. Mo-MDSCs, on the other hand, mediate T-cell suppression through the induction of high levels of NO/inducible nitric oxide synthase (iNOS), suppressive cytokines, and prostaglandin (PG)E_2_ [27, 28, 37, 47]. Both populations are able to express the immunosuppressive enzyme ARG1 [4]. The phenotypes and definitions of MDSCs comprise an important field discussed in several other excellent reviews [2, 4–6, 27, 28, 48, 49].

## GENERAL ORIGIN OF MDSCs

**Figure 2:**
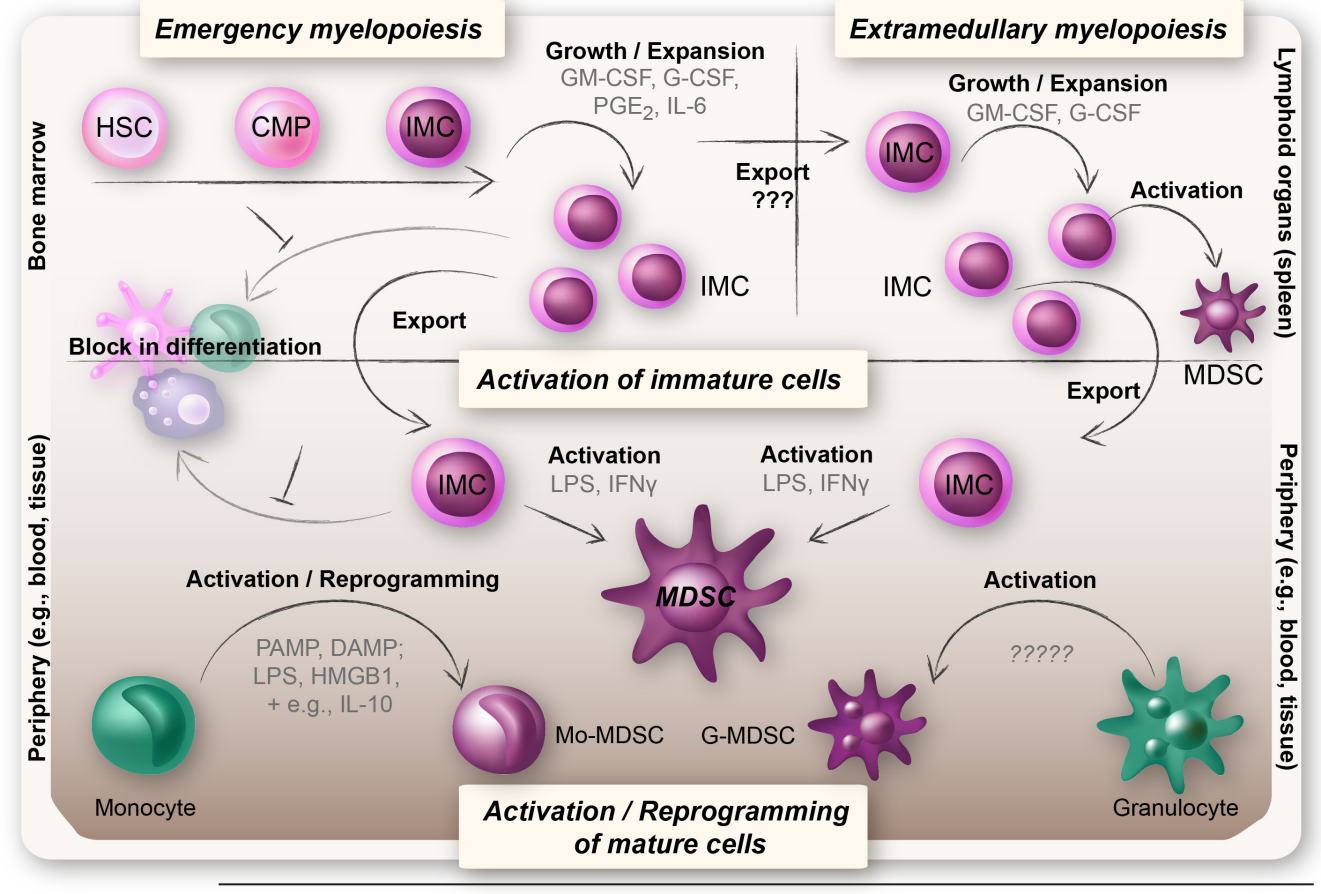
Overview of the theories on the origin of MDSCs MDSCs arise under pathological conditions when there is an excessive inflammation. Upper left panel: Upon an increased demand for myeloid cells, IMCs expand in the bone marrow and migrate into the periphery, a process known as emergency myelopoiesis. The IMCs are believed to be blocked in their differentiation and to become functionally active MDSCs when exposed to inflammatory mediators (upper and lower panel). Upper right panel: IMCs may also expand and become functionally active MDSCs extramedullary (i.e. in organs outside of the bone marrow, foremost the spleen), a feature often seen in chronic inflammatory diseases such as cancer. Lower panel: More recent hypotheses suggest that Mo-MDSCs and G-MDSCs may represent reprogrammed or activated monocytes and granulocytes. Reprogramming of monocytes into Mo-MDSCs is believed to rely on a repeated TLR-signaling (triggered by PAMPs or DAMPs) in combination with certain cytokines and mediators (e.g., IL-10, Wnt5a, and PGE2), whereas G-MDSCs are thought to represent an activation state of neutrophils.

MDSCs are important for immune suppression in cancer patients and thereby also pose a major obstacle that needs to be overcome for successful anti-cancer immunotherapy treatments. Furthermore, MDSCs decrease after tumor resection and therefore the generation and maintenance of MDSCs appears to be an active process that is nourished by tumor cells [50, 51]. An important issue concerning MDSC generation to remember is that tumors do not invent new biology, they highjack existing mechanisms. To break this vicious cycle in which MDSCs are generated, and to target MDSCs to enhance the effects of cancer therapies, it is important to understand the origin of MDSCs. See Figure 2 for an overview of the theories on the origin of MDSCs.

## ABNORMAL MYELOPOIESIS

All MDSCs undoubtedly derive from common myeloid progenitors and their development is likely governed by the same growth factors that control normal myelopoiesis e.g., granulocyte-macrophage colony-stimulating factor (GM-CSF), granulocyte colony-stimulating factor (G-CSF), and macrophage colony-stimulating factor (M-CSF) [52–58]. MDSCs arise under pathological conditions, possibly as a result of a persistent signal of low strength coming from tumors or chronic inflammation [48, 59]. However, the strength-of-signal hypothesis might be difficult to defend for two reasons. First, the frequency of MDSCs positively correlates with clinical stage and tumor burden in different cancers [13–17] and second, MDSCs are also found in patients with overt, acute conditions such as trauma and sepsis [13, 60, 61]. Most likely, it is the persistent mixture or combination of signals that generate MDSCs. Among these signals, the CSFs seem to have a prominent role.

## THE INVOLVEMENT OF CSFs IN MDSC GENERATION

**Figure 3:**
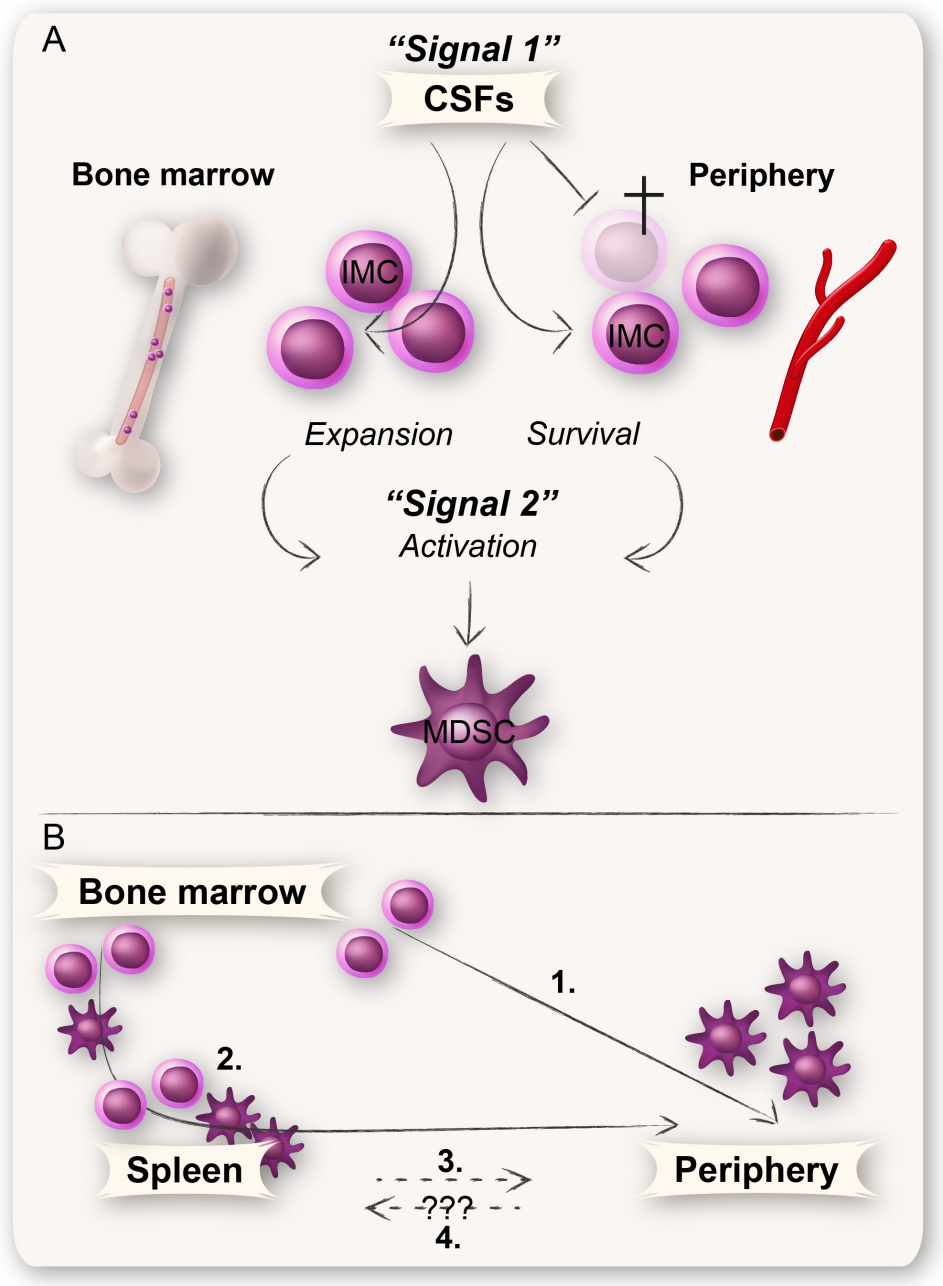
A schematic illustration of the involvement of CSFs in the generation of MDSCs **A:** MDSC development is likely governed by the same growth factors that control normal myelopoiesis e.g., CSFs (GM-CSF, G-CSF, and M-CSF). CSFs are essential survival factors for myeloid cells, in the bone marrow as well as in the periphery. They are also involved in many aspects of MDSC generation such as expansion of IMCs in the bone marrow, migration of myeloid cells into the periphery, and in some instances also for activation of MDSCs. **B:** All MDSCs undoubtedly derive from common myeloid progenitors in the bone marrow. The IMCs either expand in and migrate from the bone marrow into the periphery where they attain their MDSC phenotype (1), or into the spleen where they expand and become activated MDSCs that further migrate into the periphery (2). IMCs or MDSCs derived from hematopoietic progenitor cells found in the spleen may also directly migrate into the periphery (3). Whether peripheral MDSCs also can migrate into the spleen is currently unknown (4).

It is widely debated whether the disease-dependent generation and expansion of MDSCs occurs in the bone marrow, periphery, or extramedullary predominantly in the spleen. MDSCs have been found in both the bone marrow and spleen of humans and mice [62–64]. The fact that MDSCs from the bone marrow, spleen, and blood as well as from tumors and metastases share a similar surface phenotype supports the notion that MDSCs have a common ancestor [49, 65]. There are several tumor-derived factors that could affect the myelopoiesis both in the bone marrow and extramedullary; the best described are GM-CSF, G-CSF, and M-CSF (Figure 3) [66].

GM-CSF, G-CSF, and M-CSF are hematopoietic growth factors that play an essential role in recruitment, proliferation, and maturation of granulocytes and macrophages. These growth factors are also crucial for the survival of myeloid cells [67–69]. Several in vitro studies have shown that bone marrow precursor cells treated with G-CSF or GM-CSF acquire a surface phenotype similar to MDSCs found in blood of cancer patients [70–73].

The administration of G-CSF and GM-CSF is a common therapy for a variety of disorders. In cancer, G-CSF and GM-CSF are often used as an adjuvant to ameliorate neutropenia and to limit the extent of infections due to neutropenia [74, 75]. In patients with colorectal carcinoma, rhGM-CSF was demonstrated to initiate wound healing through stimulation of angiogenesis after surgery [76]. There are, however, inconsistent results of the beneficial role of GM-CSF as adjuvant in cancer vaccines. For instance, one clinical study demonstrated that when GM-CSF was given as a low-dose adjuvant, an increase in peripheral blood MDSCs was observed in patients with stage IV metastatic melanoma [77], whereas no effect on MDSCs was seen in patients with advanced pancreatic cancer [78]. Still, GM-CSF and G-CSF are highly secreted by many cancer cells, and elevated serum levels of these cytokines have also been linked to an increase in circulating MDSCs [49, 53, 79]. In fact, tumor-derived GM-CSF affects both the generation, maintenance, and survival of MDSCs, and its constant production might be important for the large accumulation of MDSCs found in cancer patients. In contrast, the short administration of GM-CSF during clinical treatment is not believed to have the same effects on MDSC generation (Figure 3) [55]. It will be interesting to follow the ongoing clinical studies concerning the use of GM-CSF in combination with novel immune checkpoint inhibitors (e.g., anti-PD-1 [programmed cell death protein 1]), as it has been reported that myeloid cells lacking PD-1 evoke a stronger intra-cellular signal in response to GM-CSF [80]. Whether this response is beneficial for the generation of functionally mature myeloid cells, MDSCs, or both, remains to be seen.

GM-CSF, G-CSF, and M-CSF appear to affect the generation of Mo-MDSCs and G-MDSCs differently. It was shown early on that GM-CSF and M-CSF could generate suppressor cells from the bone marrow with different phenotypes. GM-CSF induced indomethacin-sensitive suppressor cells that release high levels of PGE_2_, whereas M-CSF gave rise to indomethacin-insensitive suppressor cells with low PGE_2_ release [81]. It is known that GM-CSF affects Mo-MDSC- and G-MDSC generation and mobilization from the bone marrow [54], whereas G-CSF induces the accumulation and suppressive functions of G-MDSCs [82] and M-CSF is important for the generation and immunosuppressive functions of Mo-MDSCs [83]. It is therefore not surprising that changes in expression levels of these mediators, either via infusion as a treatment option, peripherally in a primary tumor, or in the bone marrow as a result of bone metastases, would affect the generation of Mo-MDSCs and G-MDSCs differently [54].

## EMERGENCY MYELOPOIESIS

In cancer and infections, elevated levels of CSFs induce emergency myelopoiesis to meet the increased demand for myeloid cells [55, 84–86]. Emergency myelopoiesis produces myeloid cells that migrate from the bone marrow, before they are fully mature, in response to inflammatory signals in order to renew or restore the peripheral populations that are consumed [67, 87]. Pathological conditions such as cancer and sepsis may invoke a prolonged and marked expansion of immature myeloid cells (IMCs) in the bone marrow, which eventually migrate into the blood stream where they become functionally active MDSCs with suppressive properties (Figure 2) [4, 5, 48, 88–91].

**Figure 4:**
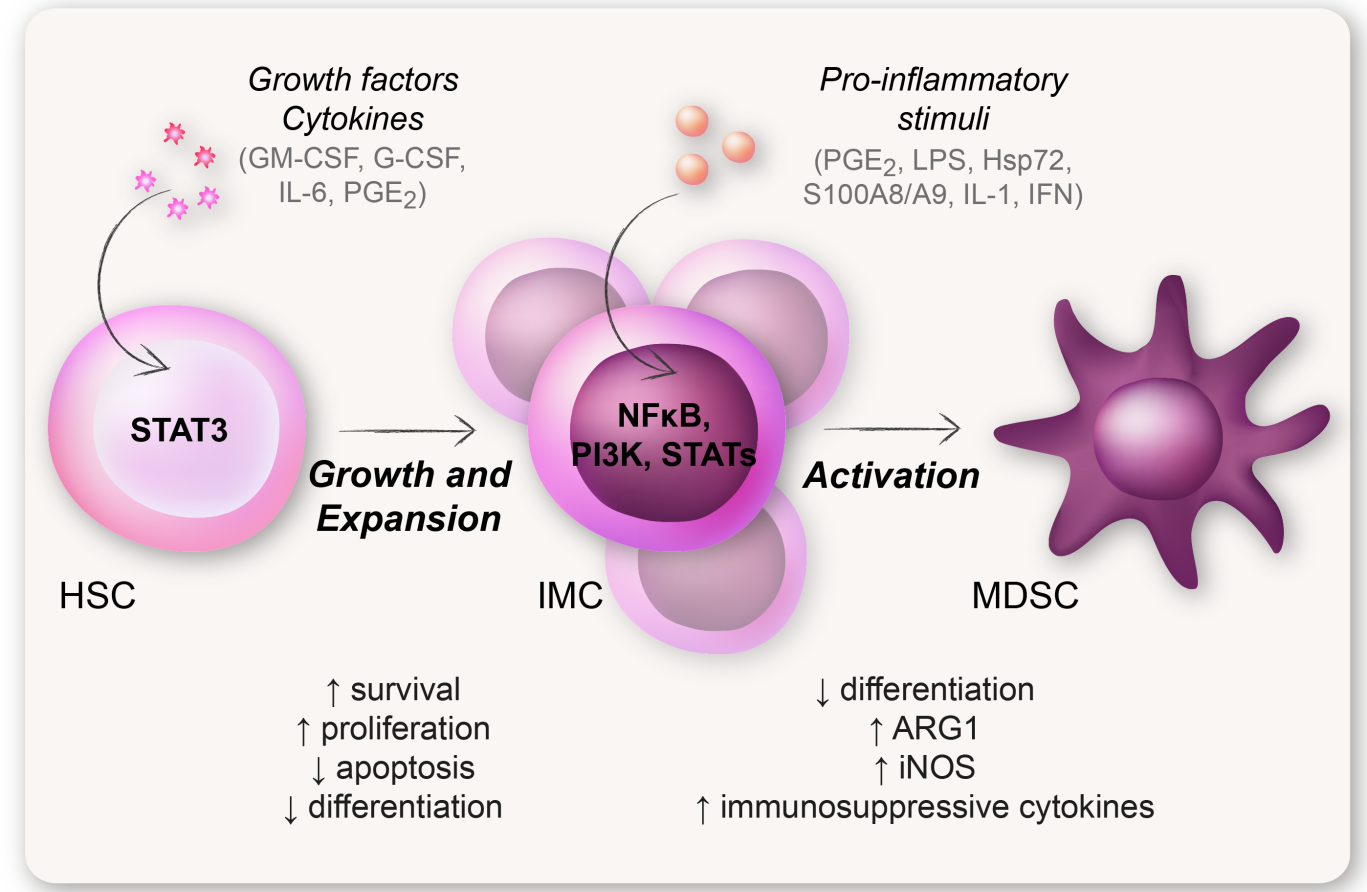
The two-signal model of MDSC generation The classical hypothesis regarding the generation of MDSCs is the “two-signal model”. This model states that an expansion signal induced by e.g., CSFs (such as GM-CSF and G-CSF), IL-6, and PGE2, and mediated by STAT3, expands and mobilizes immature myeloid cells from the bone marrow. This is followed by an activation signal induced by pro-inflammatory stimuli e.g., LPS, PGE2 and S100A8/A9, and mediated by NFκB. According to this hypothesis it is not until the IMCs acquire an activation signal that they obtain a suppressive MDSC-phenotype.

The classical hypothesis governing the molecular mechanism behind MDSC generation today is the “two-signal model” proposed by Gabrilovich et al. (Figure 4) [4]. This model states that an expansion signal 1, mediated mainly by STAT3 (induced by e.g., GM-CSF, G-CSF, and IL-6), mobilizes the IMCs from the bone marrow. This is followed by an activation signal 2, mediated mainly by the transcription factor NFκB (induced by pro-inflammatory stimuli e.g., TLR signaling and cytokines) (Figure 4) [4, 48]. However, Chalmin et al. demonstrated that the expansion of IMCs was induced by tumor-derived GM-CSF, but was not dependent on STAT3 activation. The activation of MDSCs, on the other hand, was induced by heat shock protein 72 on tumor-derived exosomes that triggered TLR2-NFκB signaling, with a subsequent production of IL-6 and activation of STAT3 [92]. This study highlights the fact that STAT3 might be implicated in several stages in the generation of MDSCs, and that many factors are involved and cooperates in the expansion and activation of MDSCs [92].

Recently, another critical transcription factor of downstream of GM-CSF, M-CSF, and G-CSF was shown to drastically affect the generation of both MDSCs and anti-inflammatory macrophages (M2), namely RORC1 (retinoic-acid-related orphan receptor C1). RORC1 was shown to drive cancer-induced emergency myelopoiesis by affecting other crucial transcription factors regulating myelopoiesis, e.g., C/EBPβ (CCAAT-enhancer-binding protein-β) and SOCS3 (suppressor of cytokine signaling 3), acting downstream of the CSFs. It is likely that both RORC1 and STAT3, together with NFκB, are important inducers of MDSC generation and expansion [48, 93].

Another study demontrated increased hematopoiesis in mice with IL-1β-secreting tumors, shown by splenomegaly, leukocytosis, and anemia [64]. The increased hematopoiesis was found in the bone marrow, where IL-1β stimulated the release of IMCs into the blood. The IMCs then migrated into the spleen where they proliferated and attained their suppressive phenotype [64]. This study indicated an increased emergency myelopoiesis in the bone marrow of mice, giving rise to increased IMCs in the blood. The IMCs then migrated into the spleen, where extramedullary myelopoiesis occurred, and received a second signal for activation [64]. Long before this article was published, Young et al. demonstrated a similar phenomenon with an increased amount of IMCs in blood, and an elevated hematopoiesis in the bone marrow and spleen in mice with metastatic Lewis lung carcinoma tumors [94]. These two studies demonstrate that the expansion of MDSCs may occur not only in the bone marrow, but also in peripheral organs or tumors.

## EXTRAMEDULLARY MYELOPOIESIS

Extramedullary myelopoiesis is defined as myelopoiesis occurring in organs outside of the bone marrow and is frequently seen in chronic inflammatory diseases, cancer, trauma, and sepsis [95]. In mice, a fraction of hematopoietic stem and progenitor cells has been shown to migrate out of the bone marrow into the blood and further out into peripheral tissue and lymph vessels [96]. Tumor-derived factors might be responsible for the migration of precursor cells into the peripheral tissue in an emergency myelopoiesis manner [64]. The progenitor cells would then proliferate and become MDSCs through activation at extramedullary sites (Figure 2). In this model, the activation of TLRs appears to be central for the generation of MDSCs. For instance, LPS has been demonstrated to induce the accumulation of MDSCs at extramedullary sites, especially the spleen [96, 97]. Although LPS alone appears to have the ability to generate MDSCs, it seems there is an even more powerful generation of MDSCs with the right cytokine combination (e.g., IFN γ) [60, 98].

It has been shown that c-kit+ hematopoietic precursors are increased in the spleen of tumor-bearing mice, a strong indication that increased extramedullary myelopoiesis does occur [52]. The generation of MDSCs from c-kit+ hematopoietic precursors in that study was dependent on GM-CSF [52]. Whether the increase of these hematopoietic precursors is directly connected to the simultaneous increase of MDSCs at the extramedullary sites in vivo is not fully proven. There are many indications that extramedullary myelopoiesis might be a consequence of an emergency myelopoiesis that induces the migration of IMCs out of the bone marrow into the periphery, where they then accumulate in the spleen and get their second signal to become MDSCs [64, 94]. However, during embryogenesis, before the hematopoiesis is established in the bone marrow, hematopoietic elements from the yolk sac are circulating in the embryo. These hematopoietic progenitor cells accumulate in the liver, but also in the spleen. Hematopoiesis can thus take place in these organs until birth [95, 99]. The persistence of progenitor cells in the spleen after birth might also be a source for MDSCs. Suppressive Gr1+CD11b+ cells in the spleen of healthy mice have been described, suggesting that MDSCs are not only induced upon infection or inflammation but also exist in steady state [98].

The finding of extramedullary hematopoiesis comes from experiments in mice; the equivalent in humans has not been fully explored. Nonetheless, it is well documented that treatment with G-CSF and GM-CSF causes an increase in spleen size in humans [100–102] that appears to be the result of extramedullary myelopoiesis [103]. This was confirmed with histology of patients receiving G-CSF, and indicates that G-CSF and GM-CSF not only induce the expansion of MDSCs in the bone marrow of humans, but also in the spleen [104].

## BLOCK IN DIFFERENTIATION

In pathological conditions such as cancer, emergency myelopoiesis creates a prolonged and marked expansion of bone marrow-derived IMCs that migrate out into the periphery [87]. The IMCs are believed to be arrested in their immature phase by inflammatory mediators such as S100A8, S100A9, VEGF, IL-10, and COX-2/PGE_2_ [89–91, 105–107]. It has also been suggested that systemically released inflammatory mediators are unable to induce MDSCs alone, but that a direct tumor cell contact or a close proximity to tumor cells would be required for MDSCs to be generated [107]. Either way, when MDSCs are taken from the tumor environment, the block is reversed and MDSCs can differentiate into mature myeloid cells, preferably monocytes/macrophages or dendritic cell [108, 109].

Both emergency myelopoiesis and block in differentiation are linked to an abnormal and persistent activation of STAT3, and many of the mediators involved in emergency myelopoiesis are also responsible for arresting IMCs in their immature phase [4]. The block in differentiation of IMCs might be an indirect effect of the tumor-derived mediators initially responsible for emergency myelopoiesis. For example, activating STAT3 in myeloid progenitor cells leads to the induction of S100A8 and S100A9 expression, which subsequently acts in an autocrine manner to arrest the cells in their immature phase (Figure 2) [4, 90].

As a treatment strategy, many studies have attempted to force the differentiation block of MDSCs to minimize the accumulation and immunosuppressive effects of these cells, in different diseases [41, 109–113]. One example is ATRA (all-trans-retinoic acid), a compound that is structurally similar to vitamin A and used to treat various malignancies. ATRA has been demonstrated to reverse the differentiation block of MDSCs and to enhance the maturation of these cells in humans [41, 109, 110]. Further studies have led to the identification of another vitamin, vitamin D3, with similar effects. In the presence of 1α,25-dihydroxyvitamin D3 the differentiation of CD34+ MDSCs into phenotypically and functionally DC-like cells in vitro was accelerated [111]. In addition, the number of circulating CD34+ MDSCs was reduced in patients with head and neck squamous cell carcinomas receiving 25 hydroxyvitamin D3 [112]. In a chronic inflammation model the use of a TNFα antagonist reversed the block in differentiation and augmented the maturation of dendritic cells and macrophages [113]. In addition, the blockage of the S100A8/S100A9 receptor on MDSCs with a carboxylated-N-glycan-specific antibody reduced the number of circulating MDSCs in tumor-bearing mice [65]. These studies demonstrate that the accumulation of MDSCs can be targeted by inducing differentiation.

## ORIGIN OF MO-MDSCs

Although MDSCs have traditionally been viewed as immature cells, emerging evidence suggest that they are rather an intermediate or even alternative state of myeloid cell differentiation. Mo-MDSCs do not have an immature morphology, only a surface marker phenotype similar to myeloid cells of the monocytic lineage, lacking activation markers [4, 23, 114, 115]. However, Mo-MDSCs have been demonstrated to overexpress the co-receptors/activation markers CD80 and CD83 [13, 116] indicating that Mo-MDSCs are indeed not as immature as previously thought. Also, Mo-MDSCs are characterized, among other things, by their expression of CD14 [23, 37], which signifies lineage commitment. This contradicts the theory that Mo-MDSCs have their origin in emergency myelopoiesis, and there is no firm proof that Mo-MDSCs are generated in this way. Most of the studies confirming the existence of emergency myelopoiesis demonstrated results for MDSCs in general [64, 92] and the dominance in number of G-MDSCs relative to Mo-MDSCs may conceal the true nature of Mo-MDSC generation. Instead, Mo-MDSCs might originate from monocytes. This hypothesis is further strengthened by the fact that Mo-MDSCs have been shown to differentiate into tumor-associated macrophages in tumors [117], a finding that could simply represent the migration of the Mo-MDSCs into the tumor.

## REPROGRAMMING OF MONOCYTES

**Figure 5:**
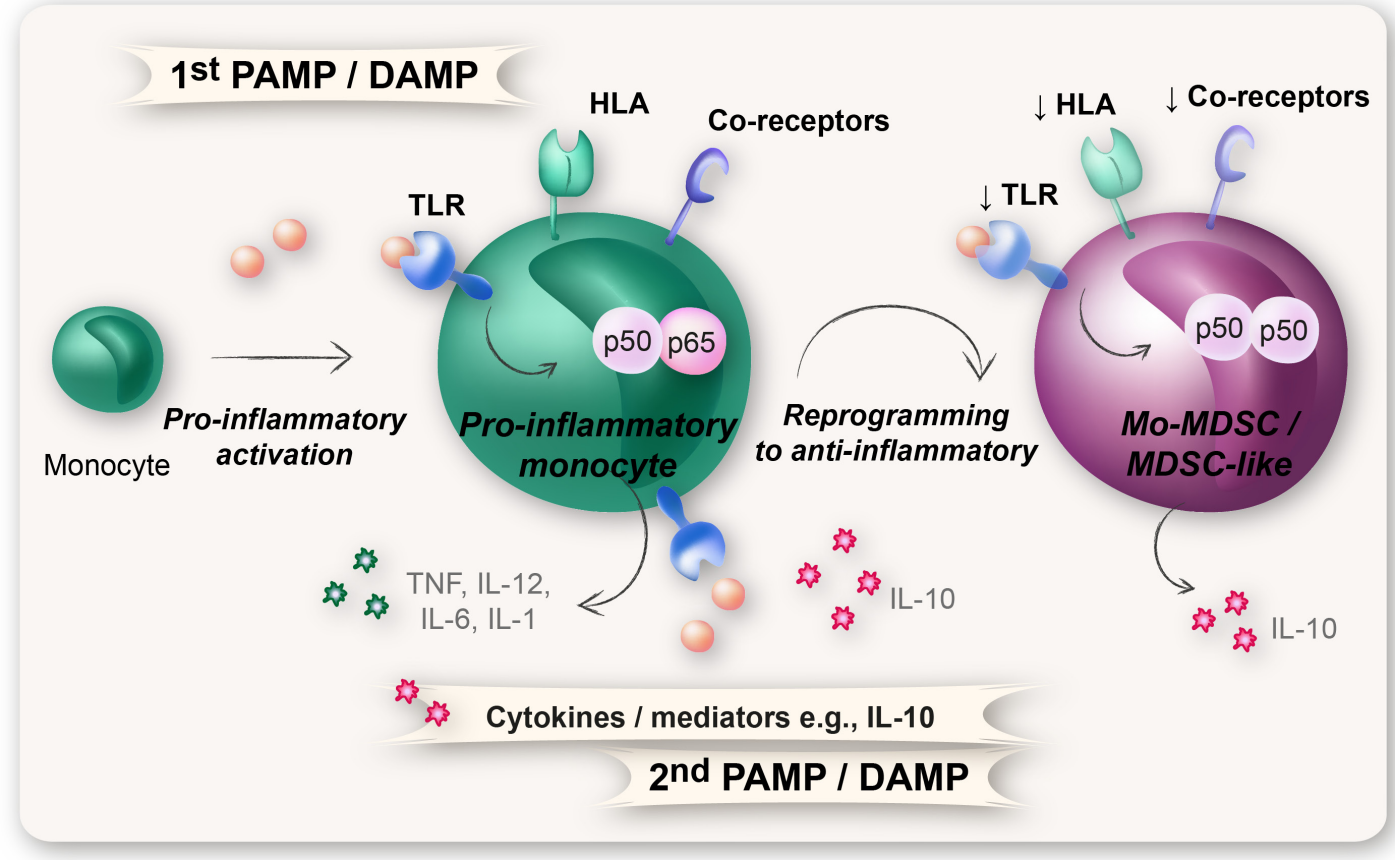
Reprogramming of monocytes into Mo-MDSCs The molecular mechanism behind reprogramming of monocytes into Mo-MDSCs is suggested to rely on a repeated TLR-signaling in combination with the right cytokine milieu (e.g., IL-10). Upon a first encounter with PAMPs or DAMPs, the monocytes will attain an activated pro-inflammatory phenotype with formation of pro-inflammatory NFκB p65:p50 heterodimers. A subsequent exposure to PAMPs/DAMPs, in combination with certain cytokines (e.g., IL-10), results in the formation of immunosuppressive NFκB p50:p50 homodimers and a reprogramming of monocytes from a pro-inflammatory state to an anti-inflammatory state with down-regulation of TLR, HLA-DR, and co-receptors. These anti-inflammatory monocytes have the same surface phenotype and function as Mo-MDSCs.

Myeloid cells with a Mo-MDSC phenotype have been demonstrated to originate from monocytes that acquire a suppressive phenotype under certain inflammatory conditions. One example of this is the endotoxin tolerance in sepsis patients where a subsequent dose of endotoxin, together with the right cytokines, results in a reprogramming of monocytes from a pro-inflammatory state to an anti-inflammatory state (Figure 5) [26, 118, 119]. These reprogrammed, anti-inflammatory monocytes (CARS-monocytes; compensatory anti-inflammatory response-monocytes) have the same surface phenotype (CD14+HLA-DR-/lowCo-receptor-/low) and function as Mo-MDSCs [26, 118, 119]. Such reprogramming can be regarded as an alternative differentiation, when the normal differentiation pathway is circumvented and the cell achieves another function. Under normal circumstances, monocytes differentiate into macrophages or dendritic cells. However, in conditions of excessive inflammation such as trauma and sepsis, where the proper cytokine milieu is created, monocytes can be reprogrammed and become Mo-MDSCs to limit the devastating effect of an inflammatory response [26, 118, 119]. This phenomenon was recently proposed to apply in cancer patients as well [13].

In a recent study, we showed that the gene expression profile of Mo-MDSCs from breast cancer patients was significantly more similar to that of reprogrammed anti-inflammatory monocytes from sepsis patients than to monocytes isolated from either healthy donors or tuberculosis patients [13]. The molecular mechanism behind monocyte reprogramming, in sepsis as well as in cancer, is suggested to rely on a repeated TLR-signal in combination with certain cytokines or mediators (e.g., IL-10, Wnt5a, and PGE_2_) thus leading to the formation of immunosuppressive NFκB p50:p50 homodimers instead of pro-inflammatory NFκB p65:p50 heterodimers (Figure 5) [13, 26, 120–122]. TLR-ligands (foremost LPS) are known to induce the expansion and activation of MDSCs [123]. In cancer, the TLR-ligands responsible for this are damage-associated molecular patterns (DAMPs; endogenous alarmin molecules), whereas in sepsis the TLR-ligands are mainly composed of the exogenous molecules pathogen-associated molecular patterns (PAMPs, such as LPS) [124]. In a way, monocyte reprogramming could be viewed as a “two-signal model,” again involving both STAT3 and NFκB, but with the difference that monocytes are affected.

Other reports also confirm that monocytes can be reprogrammed into Mo-MDSCs by demonstrating that peripheral blood monocytes can serve as precursors to Mo-MDSCs under specific conditions in vitro [106, 107, 120, 125, 126]. This further indicates that monocytes can indeed be the source of Mo-MDSCs. For instance, tumor cell lines have been shown to convert peripheral blood monocytes into Mo-MDSC-like cells with T-cell suppressive functions [107, 125]. Furthermore, tumor-derived PGE_2_ was demonstrated to drive the differentiation of monocytes towards Mo-MDSCs instead of dendritic cells in the presence of GM-CSF and IL-4 in vitro [106]. These PGE_2_-induced Mo-MDSCs resembled patient-derived Mo-MDSCs in phenotype and suppressive functions [127].

## ORIGIN OF G-MDSCs

G-MDSCs represent a heterogeneous population of cells with regards to nuclear morphology, ranging from blasts to PMNs [29, 128]. Whether the different cells within the G-MDSC-population all have a bona fide MDSCs function is unknown. Hence, it is not known whether the MDSCs with a PMN-morphology resemble neutrophils, or whether the blasts are just immature cells with no distinct function, or indeed functional MDSCs [29, 128–130]. The blast population might actually, functionally, represent the true G-MDSCs.

G-MDSCs are isolated from the mononuclear cell fraction of Ficoll density gradients and defined based on their granulocytic scatter profile (FSC/SSC) and surface phenotype using flow cytometry [29]. However, degranulated neutrophils, after activation, have also been seen to co-purify with mononuclear cells in density gradients [131]. Such degranulated neutrophils correlated with an increased level of serum ARG1 in patients with glioblastoma multiforme [131]. This implies that G-MDSC function, correlating with ARG1-mediated T-cell suppression in humans, might simply be mediated by activated neutrophils.

## ACTIVATION OF NEUTROPHILS

The theory regarding whether G-MDSCs are activated granulocytes is controversial; as is the question of whether these cells arise in the periphery or in the bone marrow. The hypothesis that either immature or mature cells can attain a suppressive phenotype in the bone marrow was termed “suppressive granulopoiesis”, and this process was shown to be driven primarily by G-CSF [29]. Indeed, given the plasticity of neutrophils, G-MDSCs could be a functionally heterogeneous subsets of neutrophils [29].

Neutrophils were until recently thought to consist of one population, but accumulating evidence suggests the existence of distinct neutrophil subsets with diverse roles in infection, inflammation, and cancer [132–136]. Pillay et al. recently identified three distinct neutrophil subsets during acute systemic inflammation in humans based on the expression of CD16 and CD62L: CD16dim CD62Lhigh, CD16high CD62Lhigh, and CD16high CD62Ldim [134]. These subsets are thought to represent different stages of neutrophil activation. The same work demonstrated that the activated neutrophils (CD16high CD62Ldim) inhibited T-cell responses through cell-to-cell interactions via macrophage-1 antigen (Mac-1; consisting of CD11b and CD18) [134]. Neutrophils are known to interact with, and modulate, T-cell responses. Many of the molecules that inhibit T-cell responses such as ARG1 and ROS are present in both activated neutrophils and G-MDSCs [137], and at least ROS has also been shown to have a direct anti-tumor function by inducing tumor cell lysis [138, 139]. The activation of neutrophils in vitro by inflammatory cytokines such as GM-CSF, G-CSF, TNF, IL-1β, and IFN- γ has been shown to prolong the survival of neutrophils [140]. A longer lifespan could allow neutrophils to carry out more complex activities with regulatory functions.

Neutrophils have been ascribed both anti- and pro-tumorigenic functions (N1 and N2 neutrophils, respectively). Their different functions might depend on the microenvironment, whether it is an acute or chronic inflammation, the cells with which they co-operate, and their activation status per se [137, 141]. This illustrates the plasticity and heterogeneity of neutrophils, which also might explain the neutrophil “immunogenic switch” theory, where a switch from an anti-tumorigenic to a pro-tumorigenic immune phenotype is proposed to occur during tumor progression [135, 136, 142].

Neutrophils are very reactive cells that are easily activated in vitro. This, together with their relatively short half-life, makes them difficult to study in their native state and the findings may therefore be biased [29, 137]. Many of the studies of neutrophils have been performed in mice in vivo. The role and function of tumor-infiltrating myeloid cells, especially TANs, in humans may be different compared to mice [130, 136]. Indeed, mice have shorter life-spans and mouse tumor models are characterized by high tumor burden and rapid tumor growth. In contrast, human tumors evolve over years to decades indicating obvious differences in tumor characteristics. In addition, neutrophils are more abundant in humans compared to mice [130, 143]. Caution is therefore called for in the process of defining this heterogeneous cell population. Because neutrophils and G-MDSCs share similar granulocytic morphology and surface phenotype, and that there is no clear consensus on how to distinguish between them, it is difficult to make definite conclusions regarding the relationship between neutrophils and G-MDSCs. This issue thus merits further investigation.

## CONCLUSIONS

MDSCs are regulatory cells with the ability to limit the extent of inflammation and to initiate wound healing and recovery. An excessive inflammatory response, such as in sepsis, will induce a signal to initiate an anti-inflammatory response and generate MDSCs. Similarly, cancer can be regarded as a site of chronic inflammation where the induction of anti-inflammatory MDSCs can be seen as a regulatory mechanism to dampen inflammation and to induce wound healing mechanisms. Indeed, cancer is not an inventor: it is an opportunist. As such, cancer exploits the simple homeostatic mechanism of MDSC generation to avoid immune surveillance and to promote tumor growth and metastasis.

A great number of MDSC-phenotypes have been described, but it is not only the surface phenotype that differs between different cancers. There are also variations in mechanisms of suppressions and in nuclear morphology. These dissimilarities in phenotype, morphology, suppressive capacity, and mechanisms might emerge from differences in origin or from different activations. Hence, Mo-MDSCs and G-MDSCs might be generated in distinct ways. Emerging evidence suggest that Mo-MDSCs are generated by a reprogramming of monocytes into Mo-MDSCs, whereas G-MDSCs might be a phenotype of neutrophils generated through the activation of immature or mature granulocytes and thereby merely represent different stages of activation. The ways in which Mo-MDSCs and G-MDSCs are generated may be distinct, but CSFs, STAT3, and NFκB seem to be central molecular players and as such may lead to new discoveries on how to target both MDSC-subtypes simultaneously. New factors that are important for the establishment of MDSCs are being unraveled, but there are still more to discover. It is very important to understand how MDSCs are generated and which factors are involved in the process to know how to design future MDSC-targeting therapy. To target MDSCs and the anti-inflammatory response might increase immunosurveillance and help improve overall survival in cancer patients.
